# Extended Reality Head-Mounted Displays Are Likely to Pose a Significant Risk in Medical Settings While Current Classification Remains as Non-Critical

**DOI:** 10.3390/microorganisms12040815

**Published:** 2024-04-17

**Authors:** Adrian Goldsworthy, Matthew Olsen, Andy Koh, Thibaut Demaneuf, Gobinddeep Singh, Reem Almheiri, Brendan Chapman, Shaima Almazrouei, Rose Ghemrawi, Abiola Senok, Simon McKirdy, Rashed Alghafri, Lotti Tajouri

**Affiliations:** 1 Harry Butler Institute, Murdoch University, Perth, WA 6150, Australia; agoldswo@bond.edu.au (A.G.); brendan.chapman@murdoch.edu.au (B.C.); s.mckirdy@murdoch.edu.au (S.M.); r.alghafri@dubaipolice.gov.ae (R.A.); 2 Faculty of Health Sciences and Medicine, Bond University, Robina, Gold Coast, QLD 4226, Australia; molsen@bond.edu.au (M.O.); gobinddeep.singh@student.bond.edu.au (G.S.); 3 Department of Forensic Medicine, Kindai University, Osaka 589-8511, Japan; andy.koh@med.kindai.ac.jp; 4 The Pacific Community (SPC), Noumea 98848, New Caledonia; thibautd@spc.int; 5 Dubai Police Scientists Council, Dubai Police, Dubai, United Arab Emirates; ralootah@dubaipolice.gov.ae; 6 International Centre for Forensic Sciences, Dubai Police, Dubai, United Arab Emirates; frn.srashed@dubaipolice.gov.ae; 7 Health and Biomedical Research Center, College of Pharmacy, Al Ain University, Al Ain, United Arab Emirates; rose.ghemrawi@aau.ac.ae; 8 AAU Health and Biomedical Research Center, Al Ain University, Abu Dhabi, United Arab Emirates; 9 College of Medicine, Mohammed Bin Rashid University of Medicine and Health Sciences, Dubai, United Arab Emirates; abiola.senok@mbru.ac.ae; 10 School of Dentistry, Cardiff University, Cardiff CF10 3AT, UK

**Keywords:** extended reality, virtual reality, augmented reality, fomite, trojan horse, third hand, conjunctivitis, ultraviolet-C, sanitisation

## Abstract

Extended reality (XR) devices, including virtual and augmented reality head-mounted displays (HMDs), are increasingly utilised within healthcare to provide clinical interventions and education. Currently, XR devices are utilised to assist in reducing pain and improving psychological outcomes for immunocompromised patients in intensive care units, palliative care environments and surgical theatres. However, there is a paucity of research on the risks of infection from such devices in healthcare settings. Identify existing literature providing insights into the infection control risk XR HMDs pose within healthcare facilities and the efficacy of current infection control and cleaning procedures. Three databases (PubMed, Embase and CINAHL) in addition to Google Scholar were systematically searched. A total of seven studies were identified for this review. Microorganisms, including pathogenic bacteria (e.g., *Staphylococcus aureus* and *Pseudomonas aeruginosa*), were found to be present on XR HMDs. Published cleaning and infection control protocols designed to disinfect XR HMDs and protect users were heterogeneous in nature. Current cleaning protocols displayed varying levels of efficacy with microbial load affected by multiple factors, including time in use, number of users and XR HMD design features. In healthcare settings, fitting XR HMDs harbouring microorganisms near biological and mucosal entry points presents an infection control risk. An urgent revision of the Spaulding classification is required to ensure flexibility that allows for these devices to be reclassified from ‘Non-critical’ to ‘Semi-Critical’ depending on the healthcare setting and patient population (surgery, immunocompromised, burns, etc.). This review identified evidence supporting the presence of microorganisms on XR HMDs. Due to the potential for HMDs to contact mucosal entry points, devices must be re-considered within the Spaulding classification as ‘Semi-critical’. The existence of microbial contaminated XR HMDs in high-risk medical settings such as operating wards, intensive care units, emergency departments, labour and delivery wards and clinical areas with immunosuppressed patients requires urgent attention. Public health authorities have a duty of care to develop revised guidelines or new recommendations to ensure efficient sanitation of such devices.

## 1. Introduction

### Overview

Extended reality (XR), encompassing virtual, augmented and mixed reality, is increasingly being integrated across various industries from leisure and gaming to education and healthcare. In 2023, there were approximately 171 million virtual reality (VR) users globally, with healthcare anticipated to experience the greatest adoption, increasing from a market value of USD 3.11 billion in 2023, to USD 25.22 billion by 2030 [1,2]. Currently, XR head-mounted displays (HMDs) are rapidly being integrated into healthcare settings, which require strict infection control policies and procedures such as surgery [3,4,5], intensive care units [6,7,8], palliative care [9] and oncology [10,11,12,13] to improve clinical outcomes. As a result, XR equipment meets the current definition of a medical device according to the Food and Drug Administration (FDA) [14] and the Therapeutic Goods Administration (TGA) [15,16]. Hospitals and health care workers currently require clear guidance as to the optimal strategy to ameliorate risks of hospital acquired infections (HAIs) associated with XR equipment. 

Fomites are inanimate platforms subject to contact and deposition of droplets from any living organism shedding such infectious agents. Factors such as humidity, moisture, temperature, UV exposure, nature and type of microorganisms collectively influence whether microorganisms will adhere to and survive on a fomite [17,18,19]. XR equipment (1) are frequently exposed to hands, skin and biological secretions; (2) receive nasopharyngeal droplets which contain viruses and bacteria, due to close proximity to the eyes, nose and other areas of the head; (3) undergo temperature fluctuations [20], providing the suitable environmental conditions for growth of pathogens; (4) lack validated and implemented sanitation protocols and; (5) are used in high-risk healthcare settings.

Mobile phones are similar fomites which are widely utilised within the healthcare industry, and in conjunction with XR headsets as part of the visual display, to assist the healthcare provider in controlling and visualizing the XR-related intervention. Metagenomic next-generation sequencing has demonstrated mobile phones from medical staff within a paediatric intensive care, neonatal intensive care and emergency department setting have the potential to act as a reservoir for “ESKAPE” pathogens (*Enterococcus faecium*, *Staphylococcus aureus*, *Klebsiella pneumoniae*, *Acinetobacter baumanii*, *Pseudomonas aeruginosa* and *Enterobacter* spp.) and antimicrobial resistance genes [21,22]. Studies have reported that only a small quantity of microorganisms may be sufficient to infect the host [23]. Together, this research demonstrates the need to further ensure that XR HMDs, and the associated infection control risks, are managed appropriately, especially when used in high-risk patient populations and/or healthcare settings. 

The economic burden HAIs represent to already strained public healthcare systems currently represents a major global public health crisis. Worldwide costs associated with HAIs are difficult to quantify, and publicly available estimates differ between countries. In the United States of America (USA) HAIs cost an estimated USD 28–45 billion each year [24], with a recent study estimating the annual economic burden to be in the range of USD 96 billion to USD 147 billion annually, encompassing both direct and indirect costs [25]. The costs associated with HAIs fluctuates due to a series of factors, including patient population, study settings/environments, data obtained from index hospitalization costs, the inclusion of outpatient costs and multi-drug resistant infections [26,27]. 

This systematic review aims to determine if XR HMDs are potentially hazardous fomites posing a risk to individuals within the healthcare sector, and in particular, within high-risk settings and/or immunocompromised individuals. Additionally, we aim to identify and compare the recommended cleaning protocols used to date to sanitise XR equipment and to determine the efficacies of the cleaning procedures to provide recommendations for future research and to assist in the develop of clinical interventions. 

## 2. Methods

### 2.1. Search Strategy

A systematic search was developed in line with the following three-step methodological approach outlined by the Johanna Briggs Institute: (a) a preliminary literature search was undertaken in PubMed and Google Scholar, (b) additional search terms were identified and search strategies translated with the assistance of a validated search engine translation software (Polyglot, https://sr-accelerator.com/#/polyglot, accessed on 26 August 2023), (c) execution of final search strategies [28]. The search strategy consisted of: (“virtual reality” OR “augmented reality” OR “mixed reality” OR “extended reality”) AND (microbe OR microbial OR infection OR bacteria OR virus OR pandemic OR fomite) AND (Colony forming unit* OR metagenomic OR sterilis* OR clean OR disinfect* OR sanitis* OR hygiene OR guideline).

### 2.2. Information Sources and Study Selection

Three databases (PubMed, EMBASE and CINAHL) and Google Scholar were searched on 26 August 2023. All results from the databases and the first 100 Google Scholar results were exported into EndNote X9. Forward–backwards citation searching alongside an additional search for grey literature was performed in an attempt to undertake a thorough evaluation of the literature. 

### 2.3. Data Extraction and Quality Assessment of Sources

Duplicate results were removed within Systematic Review Accelerator’s validated deduplication software utilising the focused algorithm prior to being manually reviewed. Articles were screened first by title and abstract and then by full text by two authors (MO and AG) against predefined inclusion and exclusion criteria within Systematic Review Accelerator’s Screenatron. Articles which were included aimed at investigating and/or analysing the level of microbial contamination or the cleaning procedures employed. Articles were excluded if they were not available in English.

## 3. Results

### Selection of Sources of Evidence

**Figure 1:**
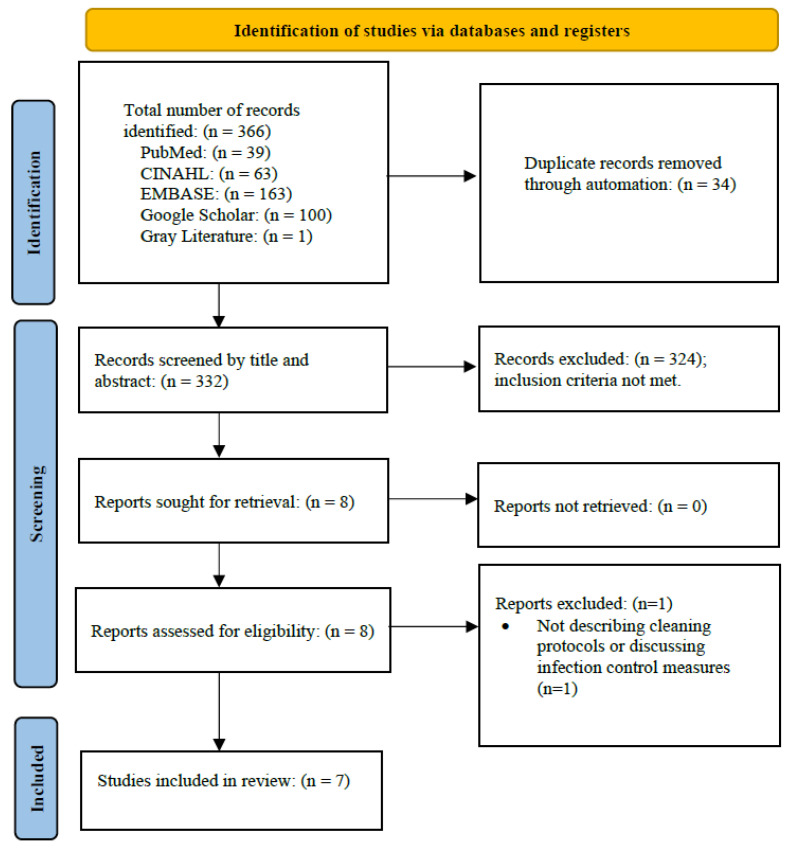
PRISMA flow diagram of selection protocol for studies included for full review.

Following the systematic search, 365 articles were identified from the literature, with 39 articles from PubMed, 163 from EMBASE, 63 from CINAHL and 100 from Google Scholar. After duplicates were removed, the remaining 331 articles were screened based on the inclusion criteria. Of these, eight full-text articles were assessed for eligibility, of which one article was excluded for not meeting the inclusion criteria. Finally, seven articles met the criteria for full review and were included in the final analysis. Figure 1 represents the PRISMA flow diagram outlining the selected studies that passed the criteria for full review.

No identified article undertook a cleaning validation of XR HMDs within a healthcare setting (Table 1). One study investigated the presence of microorganisms on devices within a university education setting [29]. Two further studies [30,31] investigated the recovery of microorganisms following inoculation of various microbes (Table 2). Two surveys [30,32] and one scoping review [6] investigated the current cleaning and infection control practices within the healthcare industry. The five cleaning protocols [29,30,31,33,34] which were described were heterogeneous, with only one article [30] explicitly advocating for cleaning to be undertaken pre and post at every utilisation of XR (Table 3).

In the study by Creel et al. [29], the most frequently isolated bacteria was *Staphylococcus aureus*, with 37 colonies reported. Additionally, this study identified antimicrobial resistance to 4 antibiotics from the 37 colonies of *Staphylococcus aureus* (Erythromycin (27 colonies), Penicillin (22 colonies), Tetracycline (24 colonies) and Gentamycin (2 colonies)) [29]. This study also identified several additional bacteria, including *Moraxella osloensis*, *Micrococcus luteus*, *Kocuria rosea*, *Rothia kristinae*, *Dermacoccus nishinomiyaensis*, *Moraxella osloensis* 2, *Corynebacterium ihumii*, *Staphylococcus argensis* and *Moraxella osloensis* 3 [29]. 

On the other hand, the study by Roberts et al. [30] performed a disinfection study where laboratory-grown bacteria were inoculated onto VR headsets. From this study, *Staphylococcus epidermidis* (ATCC 12228), *Pseudomonas aeruginosa* (laboratory strain PAO1) and *Staphylococcus aureus* (ATCC 25923) were recovered from VR headsets following disinfection. Similarly, Daniel et al. [31] performed a sterilisation study with dry chlorine dioxide (dClO_2_) with laboratory-grown bacteria inoculated onto VR headsets. From this study, *Staphylococcus aureus*, *Pseudomonas aeruginosa*, *Burkholderia multivorans*, *Acinetobacter baumanni*, *Mycobacteroides chelonae* and *Candida albicans* were inoculated, recovered and cultured from HMDs post disinfection [31]. 

## 4. Discussion

### Overview

This systematic review demonstrates that little research has been undertaken to date regarding the microbial contamination of XR devices and associated infection control procedures [6]. A wide variety of hardware devices are currently used with varying design features utilising both porous and non-porous materials [6,30,32,35]. No current “gold standard” cleaning procedures and protocols have been developed. Currently implemented cleaning strategies involve the use chemical cleaning wipes, UV-C technology, chlorine dioxide gas (dClO_2_) and reusable or disposable covers for the facial interface. In addition, no cleaning validation studies undertaken in healthcare facilities have been reported, demonstrating that XR HMDs are entirely and efficiently disinfected through robust infection control procedures. As a result, this systematic review has identified XR-HMDs as important fomites that require attention from an infection control perspective to ensure they are able to be sanitised efficiently. Our review also demonstrates a lack of scientific literature evaluating the risk current XR HMDs pose to immunocompromised individuals in health care settings.

In medical settings, XR HMDs are classified as “non-critical” medical equipment under the Spaulding classification as the device is intended to come in contact with intact skin only [33]. As a result, XR equipment is assumed to require low to intermediate levels of disinfection between patients [36]. This ‘non-critical’ classification suggests that XR devices are at low risk of infection transmission. However, this current Spaulding classification fails to consider three major points. Firstly, HMDs might not be optimally sanitised/sterilised as cleaning protocols associated with low or intermediate recommended levels of disinfection do not eliminate all non-enveloped viruses, fungi and spores, and the design of current HMDs provides significant challenges to disinfection [37]. When XR HMDs are used by patients or medical staff, the user’s flora or pathogens might be deposited on these devices’ external surfaces, crevices and optic projection areas. Liquids from microbial-laden nasopharyngeal droplets during breathing or sneezing or salivary droplets when talking or coughing are inadvertently expulsed towards the crevices, narrow fissures and adjustment-openings and will penetrate any porous surfaces on the HMDs. Additionally, XR HMDs have internal components (e.g., fans) that may become niches for pathogenic microorganisms providing additional challenges to achieving adequate levels of disinfection. Of particular interest, the study by Creel et al. [29] swabbed two HTC VIVE VR devices that were used for an Immersive Media course in the University of Mississippi. The authors showed the growth of viable microorganisms cultured in three different media with the number of colony-forming units, sourced from swabbing headsets, increasing over the course of their seven-week study [29].

Secondly, the classification fails to adequately consider the proximity of HMDs to the mucosal points of entry of the user’s exposed face as a possible means by which pathogenic microorganisms can contaminate the user via various routes. For example, during the donning and doffing of some devices, direct contact is common between the facial interface and users’ eyelids and eyelashes, structures that interface with mucous membranes of the eyes [38]. The dynamic manner in which XR HMDs are designed to be utilised may also result in microorganisms translocating from the device to various mucous membranes. Of particular consideration for healthcare settings is the manner in which XR HMDs may act as a fomite which negates hand hygiene. Only one of five cleaning protocols reported in this systematic review commented on the necessity for patients and healthcare providers to undertake hand hygiene both prior to and following XR use [30]. The adjustment and usage of suboptimally decontaminated XR HMDs by either the patient or healthcare worker negates hand hygiene procedures increasing the risk of HAIs and impact on the health of the XR users. Infections such as acute haemorrhagic conjunctivitis may result from users rubbing their eyes or face following removal of the equipment [39]. In this way, XR devices may act as ‘trojan horses’ for microbial contamination and infection spread. 

**Figure 2:**
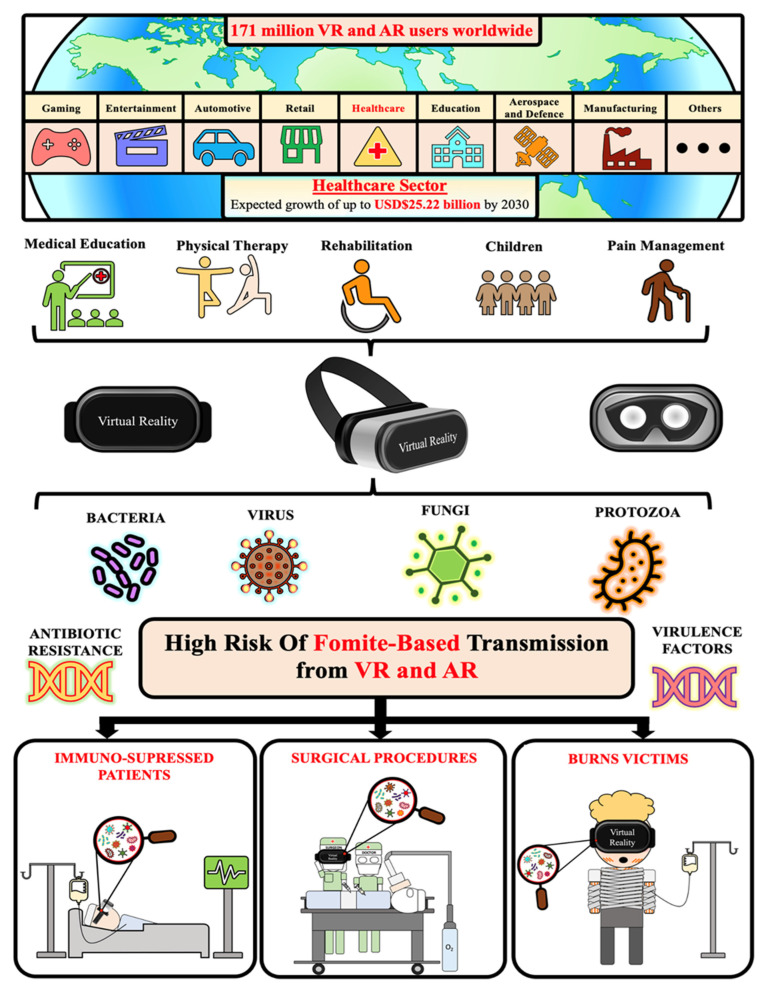
Overview of extended reality devices and their role as potential fomites in high-risk healthcare environments with implications for immune suppressed and immune-compromised individuals.

Thirdly, XR devices are increasingly being utilised within high-risk settings such as operating theatres and intensive care units as well as with immunocompromised patients (Figure 2). For example, XR headsets have been shown to have great utility and are currently utilised by both surgeons to assist in providing virtual displays and for patients to assist with pain management and anxiety during surgery. The high risks of fomite-based transmission in these settings emphasise the need for sophisticated infection control protocols and sanitisation policies to ensure these devices do not present the risks of a hazardous fomite.

Daniel et al. [31] utilised dry chlorine gas to disinfect VR HMDs. This technique may assist in disinfecting crevices and other areas that are hard to clean via other manual methods effectively. However, previous research has demonstrated dry chlorine gas to be less effective at disinfecting porous surfaces raising concern regarding the method’s efficacy at sterilising the porous surfaces associated with some facial interfaces and head straps [40]. Roberts et al. [30] also demonstrated that porous materials from such headsets were more difficult to disinfect than non-porous parts of the VR headsets. In their study, the authors investigated the efficacy of two different disinfectants (Isopropanol and quaternary ammonium) on two popular VR headsets (Oculus Quest and Oculus Quest 2). Samples were swabbed on high-touch porous and non-porous surface areas of the VR sets to monitor the survivability of three bacteria. Whilst physical barriers, both disposable and cleanable, are commonly utilised to cover the porous facial interface, no data was identified to assist in understanding if this measure is effective at preventing this porous surface from becoming a reservoir for pathogenic microbes. 

Of note, recommendations currently outlined in the draft of the international standards organisation suggest that UV-C when properly utilised is an effective cleaning tool for HMDs including their associated facial interfaces and controllers. In contrast Roberts et al. [30] describe UV-C to be an ineffective sanitisation tool for VR equipment, but details regarding devices, methodology, data and results were not disclosed. Major advantages of the use of UV-C sanitisation include the removal of human variance in cleaning efficacy as well as the ability to incorporate mechanisms to electronically record data regarding the time and date specific devices were sanitised for recordkeeping purposes. Conversely, the rapid manner in which HMDs with vastly different profiles and designs are being developed, provides challenges to the design of UV-C sanitisation devices as the light emitting diodes (LEDs) are likely required to be in close proximity to the device to be of sufficient germicidal efficacy. Further research is warranted to validate current commercially available UV-C devices as an effective sanitisation method and to facilitate the development of more effective and robust XR UV-C technology in the future.

## 5. Study Limitations

As a result of the limited literature to date which has been published investigating the microbial contamination of XR HMDs, this review is currently unable to provide strong recommendations as to the optimal strategy which should be utilised to sanitise XR HMDs. Additionally, as research investigating this important issue is published, it will likely necessitate a re-evaluation of the literature. 

## 6. Author’s Recommendations

**Figure 3:**
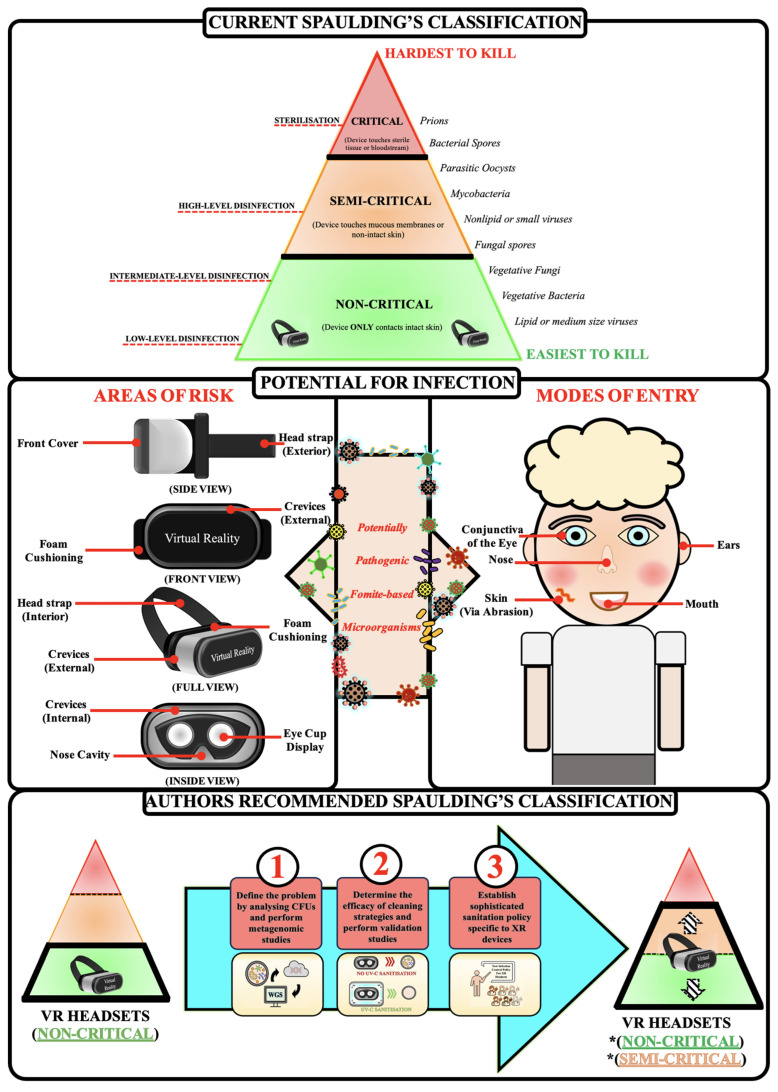
Strategic plan for further research and potential solutions to XR headset fomites. * Classification dependent on factors such as environment and patient population. For example, in physiotherapy, XR HMDs within musculoskeletal private practice should be classified as non-critical. However, when XR-HMDs are utilised in conjunction with immunocompromised individuals, they could be classified as semi-critical.

To date, very little evidence has been published describing the extent to which XR-HMDs are contaminated with pathogenic microbes. Additionally, no robust solutions are in place to mitigate these risks despite the use of XR devices in high-risk healthcare settings. Infographic 2 illustrates our recommendation for reclassifying XR equipment within the Spaulding’s classification (Figure 3). The first step to understanding the risk posed by XR equipment includes defining the microbial load and spectrum present on such devices. The characterisation of the microbial virulence and potential antimicrobial resistance present on XR equipment would further demonstrate the clinical importance of decontaminating these important fomites. The second stage involves undertaking cleaning validation studies of various cleaning protocols to validate sanitation protocols that can be effectively implemented in healthcare settings. Finally, a review of policy is required to ensure sanitisation procedures are correctly undertaken, which may also include an elaboration of Spaulding’s classification to allow for flexibility of medical devices such as XR from “non-critical” to “semi-critical” when used in high-risk environments or within various high-risk patient populations. Overall, the utility of XR devices and their benefits in the healthcare sector are paramount and the risks associated with their use from an infection control perspective must be taken seriously. 

## 7. Conclusions

The utilisation of sophisticated XR devices within the healthcare sector is becoming increasingly adopted, yet the potential for microbial dissemination from these devices is currently overlooked. HMDs are fomites, and the current application of disinfection strategies to sanitise these devices is likely to be suboptimal given the current classification as ‘non-critical’ medical devices under the Spaulding classification. The current use of HMDs within healthcare settings, especially high-risk settings such as operating theatres and with immune-compromised individuals may be exposing users to pathogenic microbes resulting in adverse patient outcomes and higher morbidity and mortality. Considering the rapid development of immersive XR HMD technology in healthcare settings and the likely fact that these devices are important fomites, based on our knowledge of other mobile devices, it is necessary to consider the re-classification of HMDs to semi-critical devices in these settings. Implementing stringent sanitisation protocols is imperative to establish effective XR infection control measures, ensuring that XR devices can continue to enhance patient outcomes within a safe patient care workflow.

## Figures and Tables

**Table 1:** Summary of aims and comments of the articles included in this systematic review.

Study ID	Article Type	Aim(s)	Comments
Moore et al., 2021 [33]	Cleaning Guideline	Aim 1: Document the process of developing an operational guide for cleaning and disinfection of HMDs in New South Wales, Australia. Aim 2: Describe the emergence of VR as a supporting modality for clinical education, the principles of infection prevention and control and how these principles underpin the development of a guideline for VR cleaning and disinfection to ensure the safety of users. Aim 3: We then identify future directions for research and innovation.	Take home messages: The proximity of VR devices to mucous membranes of the nose, mouth and eyes potentially increases risk of infection. VR HMDs are classified as “noncritical” devices under the Spaulding classification. Cleaning HMDs with ultraviolet light is likely challenging. Testing of the proposed guideline (currently in use) is required.
International Standards Organisation [34]	Guidance Document	Aim 1: Provide guidance on the safe set up and usage of VR and AR in consumer and enterprise domains.	Take home messages: In commercial settings devices should be cleaned in between each use. Users should refer to manufacturer’s recommendations regarding cleaning methods. UV-C when used properly is an effective strategy to clean XR equipment.
Høeg and Lange, 2022 [32]	Survey	Aim 1: Survey a range of stakeholders who use VR HMDs to understand the range of hygiene practices currently utilised and identify areas for future research.	Most popular VR headsets in use: Oculus Quest 2: 52% Oculus Quest 1: 38% PICO: 38% HTC VIVE: 29% Cleaning methods: Anti-bacterial wipes: 73% Alcohol disinfecting wipes: 56% Permanent face covers (leather/silicone) Confidence that their cleaning methods are sufficient: Slightly confident: 3% Somewhat confident: 26% Fairly confident: 50% Completely confident: 21% Additional notes: 81% respondents were unaware of research on hygiene practices of VR headsets. Respondents reported wanting to know more regarding effectiveness of cleaning protocols.
Goldsworthy et al., 2023 [6]	Scoping Review	Aim 1: Determine what cleaning/infection control policies/procedures are utilised for XR in paediatric intensive care units.	VR HMDs Utilised: HTC VIVE NVIS MX90 Oculus Rift Oculus Quest 1 Identified cleaning and infection control measures: Use of disposable eye masks or plastic. Use of alcohol or other germicidal disposable wipes. Periodic cleaning with UV-C wand in addition to use of chemical disinfectants after each use. Cleanliness of hardware monitored by hospital infection control department.
Roberts et al., 2022 [30]	Survey and Microbial Count	Aim 1: Determine current disinfection practices in health care settings and how they were established. Aim 2: Report on the effect of commonly used disinfectant wipes on the disinfection of VR headsets experimentally contaminated with common bacterial pathogens. Aim 3: Describe a standard operating procedure to reduce infections with multi-patient VR utilisation.	VR systems used in healthcare: Starlight Children’s VR system: 67% PlayStation VR: 56% Oculus Quest 2: 44% Google Daydream: 44% Kind VR: 11% Cleaning methods: Isopropyl alcohol: 33% Quaternary ammonium: 22% Isopropyl alcohol/quaternary ammonium: 44% Hydrogen Peroxide: 22% Physical barriers used to prevent infection: Silicon covers: 66% Disposable eye masks: 33% Wipeable replacement head straps: 22% Hair covers: 22% No barriers present: 33% Colony Forming Units key findings: Isopropyl alcohol was more effective than alcohol free quaternary ammonium to sanitise the headsets. Cleaning of porous surfaces (head straps) was less effective than non-porous surfaces. Ultraviolet light disinfection needs more research.
Creel et al., 2020 [29]	Microbial Count and rRNA sequencing	Aim 1: Analyse the potential for these headsets to become contaminated.	Take home messages: *Staphylococcus aureus* was the most identified bacteria. Staphylococcus aureus strains isolated from headsets possessed high levels of antibiotic resistance. Other identified bacteria included *Moraxella osloensis* and *Micrococcus luteus.*
Daniel et al., 2023 [31]	Microbial Count	Aim 1: Evaluate the use of dry chlorine dioxide (dCl02) gas with parametric validation as a standardised decontamination method for VR HMDs.	Take home messages: Following inoculation with *S. aureus*, *P. aeruginosa*, *B. multivorans*, *A. baumannii*, *M. chelonar* and *C. albicans*, VR HMDs were exposed to 2000 ppm/h of dCLO_2_ gas. No viable organisms were isolated following culture indicating effective sterilisation.

VR: virtual reality; HMDs: head-mounted displays.

**Table 2:** Studies confirming the presence and recovery of microorganisms from VR headsets.

Study ID	Country	Setting	Population	Number of Sampled VR Headsets	Microorganism Group	Colony Growth (Viability)	DNA Extraction Kit	DNA Sequencing	Antibiotic Sensitivity Test
Creel et al., 2020 [29]	United States of America	Public lab space at the University of Mississippi	University Students	2	Bacteria	Yes	MoBio Ultra Clean Microbial DNA Isolation Kit	16S rRNA gene sequencing	Yes
Roberts, et al., 2022 [30]	United States of America	Laboratory setting	Inoculation of HMDs	2	Bacteria	Yes	No	No	No
Daniel et al., 2023 [31]	United Kingdom	Laboratory setting	Inoculation of HMDs	2	Bacteria	Yes	No	No	No

HMDs: head-mounted displays.

**Table 3:** Cleaning protocols implemented in five articles.

Cleaning Protocol Details		Roberts et al., 2022 [30]	Moore et al., 2021 [33]	Creel et al., 2020 [29]	Daniel et al., 2023 [31]	International Standards Organisation, 2023 [34]
Before XR Use	Examine the device for signs of contamination, and if no obvious signs of contamination proceed to use.	✕	✕	✕	✕	✓
	User to wash hands before use.	N/A	✕	✕	✕	†
	Patient and staff perform hand hygiene.	✓	✕	✕	✕	✕
	Fit nonporous cover over facial interface.	✓	✕	✓	✕	†
	Cover hair with surgical cap.	✓	✕	✕	✕	✕
	Fit user with face mask.	✕	✕	✓	✕	✕
	Assess device for disinfectant suitability.	✓	✕	✕	✕	✓
	Use disinfecting wipes to clean all surfaces.	✕	✕	✓	✕	✕
	Use a device-compatible, EPA-registered product List H according to the manufacturer’s instructions for use, ensuring all surfaces are saturated.	✓	✕	✕	✕	✕
After XR Use	Expose VR HMD to 2000 ppm/h of dClO_2_ gas.	✕	✕	✕	✓	✕
	Proper use of UV-C light.	✕	✕	✕	✕	✓
	Patient to perform hand hygiene.	✓	✕	✕	✕	✕
	Staff to perform hand hygiene.	✓	✓	✕	✕	✕
	Staff don appropriate PPE inclusive of nitrile gloves +/− other equipment as required by patient’s transmission-based isolation protocols (if applicable).	✓	✕	✕	✕	✕
	Remove device from patient and place on a clean disposable pad.	✓	✕	✕	✕	✕
	Remove facial interface barrier. If disposable, discard.	✓	✓	✕	✕	✕
	Clean reusable face pads with a detergent solution or wipe.	✕	✓	✕	✕	✕
	Clean hands with alcohol-based hand rub or soap and water.	✕	✓	✕	✕	✕
	Clean all visibly soiled areas with disposable wipes or paper towels.	✓	✕	✕	✕	✕
	Ensure surfaces are wet for 2–4 min following wiping with chemical disinfectant.	✕	✕	✕	✕	✓
	Clean lenses with microfiber cloth.	✕	✕	✕	✕	✓
	Use a device-compatible, EPA-registered product List H according to the manufacturer’s instructions for use, ensuring all surfaces are saturated.	✓	✕	✕	✕	✕
	Use a new wipe to clean each surface.	✕	✓	✕	✕	✕
	Allow HMD and controllers to dry.	✓	✓	✕	✕	✓
	Store device in dry space physically separated from non-disinfected devices.	✓	✕	✕	✕	✕
	Leave items to dry and store them in a clean, sealable and disposable bag.	✕	✓	✕	✕	✕
	Patient to perform hand hygiene.	✓	✕	✕	✕	✕
	Staff to perform hand hygiene.	✓	✓	✕	✕	✕

Note: ✓: cleaning protocol stage described within publication; ✕: cleaning protocol stage not described within protocol. Details of protocols are provided before and after the use of the virtual reality headset. Abbreviations: EPA: Environmental Protection Agency; VR: virtual reality; HMD: head-mounted display; PPE: personal protective equipment; N/A: not applicable due to higher cleaning standard; †: optional.

## Data Availability

Data are contained within the article.
